# Diffusion Tensor Imaging in Rat Models of Preclinical Diabetic Nephropathy: A Preliminary Study

**DOI:** 10.3389/fendo.2021.701116

**Published:** 2021-08-27

**Authors:** Xiaoyan Hu, Min Kuang, Bo Peng, Yang Yang, Wei Lin, Wenbo Li, Yinghua Wu

**Affiliations:** ^1^Department of Radiology, Chengdu First People’s Hospital, Chengdu, China; ^2^Department of Radiology, Chengdu Second People’s Hospital, Chengdu, China; ^3^Department of Radiology, Hospital of Chengdu University of Traditional Chinese Medicine, Chengdu, China; ^4^Sichuan General Practitioner Training Center, Chengdu University of Traditional Chinese Medicine, Chengdu, China

**Keywords:** diffusion tensor imaging, kidney, diabetic nephropathy, apparent diffusion coefficient, fractional anisotropy

## Abstract

**Purpose:**

This study aimed to investigate the value of diffusion tensor imaging to assess renal injury in a rat model of preclinical diabetic nephropathy.

**Methods:**

Twenty-eight male Sprague Dawley rats were divided into two groups: the normal control (NC) group of 10 rats and the diabetic nephropathy (DN) group of 18 rats. Eight weeks after diabetes induction by streptozotocin, 3.0-T magnetic resonance (MR) imaging (b = 0 and 600 s/mm^2^, 15 diffusion directions) using a 32-channel knee coil was performed. After MR imaging, we measured serum creatinine, and collected double kidney tissues for pathology. The apparent diffusion coefficients(ADC) and fractional anisotropy(FA) values of the renal cortex and medulla were calculated for all kidneys. Physiological parameters, laboratory parameters, and imaging results were compared between the two groups.

**Results:**

All DN group animals developed hyperglycemia, polyuria, and emaciation. Serum creatinine was not significantly different between the groups (*P* > 0.05). Urinary albumin at 2, 4, and 8 weeks was higher in the DN group than in the NC group but <20 µg/min (*P* < 0.05). Pathologically, renal damage in the DN rats was observed. The ADC value was significantly increased in DN animals in the cortex (1.75×10^-3^mm^2^/s),medulla(1.53×10^-3^mm^2^/s)compared with NC group(cortex, 1.52×10^-3^mm^2^/s; medulla,1.35×10^-3^mm^2^/s). The FA value was significantly reduced in DN animals in the cortex (0.21),medulla(0.25)compared with NC group(cortex,0.26;medulla,0.3).

**Conclusions:**

Increased apparent diffusion coefficients and decreased fractional anisotropy values on diffusion tensor imaging were associated with preclinical DN. Diffusion tensor imaging may be useful in early, non-invasive, quantitative detection, and therapy monitoring of DN.

## Introduction

As a serious microvascular complication of diabetes mellitus, diabetic nephropathy (DN) is one of the major causes of end-stage renal disease (1, 2) and can induce structural changes in the kidney, including tubular dilatation, thickening of the glomerular basement membrane, and nodular and diffuse glomerulosclerosis (3). However, pathological changes are not used for therapy monitoring because biopsy is invasive and prone to sampling errors. At present, the earliest clinical evidence of DN is microalbuminuria, but in preclinical DN, excretion of urinary albumin can be within the normal range, and not all patients with microalbuminuria develop DN (4). Therefore, identification of a reliable non-invasive imaging marker for monitoring the treatment and prognosis of DN is necessary. Diffusion is the random and irregular movement of molecules. It is an important physiological activity of human body and one of the transport modes of substances in the body. In normal tissue, diffusion is rarely limited. However, in the pathological state, due to the influence of various factors, the diffusion will be limited. In human tissues, the movement of water molecules varies in different directions due to the infulence of the celluar structure of the tissue. Diffusion tensor imaging (DTI) can apply motion-sensitive gradients in at least six directions to noninvasively evaluate the diffuse motion of water molecules. DTI (5, 6) is a promising non-invasive technique that can assess renal function and pathology by qualitatively and quantitatively imaging three-dimensional diffusion of water molecules. DTI can not only describe the direction of diffusion of water molecules in tissues by fractional anisotropy (FA), but also describe the displacement degree of water molecules in tissues in the direction of diffusion sensitive gradient by apparent diffusion coefficient (ADC). The characteristics of tissues and organs can be quantitatively reflected by ADC and FA (7).In recent years, more and more studies have used DTI to evaluate diabetic nephropathy, and found that it has potential clinical value (8, 9). Lu (10) have suggested that the apparent diffusion coefficient and fractional anisotropy value may be viable imaging biomarkers in DTI that can reflect the pathological progression of DN. The purpose of this study was to investigate whether the apparent diffusion coefficient (ADC) and fractional anisotropy (FA) value can be used to quantitatively evaluate renal function changes in preclinical DN and provide a non-invasive, visual, and accurate imaging method for the diagnosis of DN.

## Materials and Methods

### Diabetic Nephropathy Model Rats

Efforts such as improving comfortable feeding environment, minimizing the times of invasive procedures, pre-operation training of animals and euthanasia were made to minimize the suffering. The animal experiments were performed in accordance with the China Laboratory animal-Guideline for ethical review of animal welfare and were approved by the ethics committee of our institution (2019048).

Twenty-eight male Sprague Dawley rats weighing 498.5 ± 54.3 g(provided by Chengdu Dashuo experimental animal co.,LTD, China) were randomly divided into two groups: the diabetic nephropathy (DN) group with 18 rats and the normal control (NC) group with 10 rats. DN group used streptozotocin (Sigma, USA) to establish diabetic nephropathy model (11, 12). 500mg streptozotocin (STZ) was dissolved in 50ml sodium citrate buffer (0.1mol/L,pH4.5) (Beijing Solaibao Technology Co., LTD, China) to prepare STZ solution. The whole operation was carried out under the conditions of dark and ice bath. Because the prepared solution is very unstable, it needs to be used and prepared now, and the injection should be completed within 30 minutes. A dose of 40 mg/kg STZ solution in the DN group was injected intraperitoneally to establish the DN model after 10 weeks of high-glucose and high-fat diet (provided by Chengdu Dashuo experimental animal co., LTD, China). The NC group rats were given a normal diet. General parameters, including food and water intake, body weight, as well as 24-hour urine volume collected by metabolic cage (Purchased from Shanghai Jianyi Instrument Equipment Co., LTD, China) of all animals were monitored regularly. Serum creatinine (Nanjing Jiancheng Biological Engineering Institute, China) and urinary albumin (Nanjing Jiancheng Biological Engineering Institute, China) at 2, 4, and 8 weeks levels were quantified according to the manufaturer’s guidelines. Blood samples from the caudal vein were taken 72 hours later to measure fasting serum glucose (Jinwen, China). According to the diagnostic criteria of diabetes mellitus (13), a rat fasting serum glucose level of >16.7 mmol/L and symptoms, such as increased drinking water, diet, urine volume, and loss of weight, are indicators that the diabetes model has been established.

### MR Protocol and Data Collecting

All animals were scanned at 8 weeks after diabetes induction by using a 3.0-T scanner (Discovery MR 750; GE, USA) with a 32-channel knee coil. To restrain the animals during MR scanning, 10% chloral hydrate(2 ml/kg) (Dalian Meilun Biotechnology Co.,LTD, China) was used. There were no signs of peritonitis in the rats after treated with chloral hydrate. T1-weighted axial images were acquired: repetition time/echo time, 360/9.5 ms; field of view (FOV), 80 × 80 mm^2^; matrix, 160 × 128; number of excitations (NEX), 4; and thickness/interval, 2.0/0.2. The spin-echo DTI sequence parameters were as follows: repetition time/echo time, 4000/89 ms; FOV, 80 × 80 mm^2^; matrix, 160 × 128; NEX, 4; thickness/interval, 2.0/0.2; b-values, 0 and 600 s/mm^2^; and diffusion directions, 15. Images were analyzed by two readers blinded to the pathological and laboratory results on a post-processing workstation (AW4. 5). ADC and FA values were obtained by the two experienced radiologists by drawing six regions of interest (ROIs) with sizes of 2 to 3 mm^2^ in renal cortex and medulla. When drawing the ROI, we select multiple ROIs at multiple levels above and below the renal hilum for data measurement, and avoid artifacts and vascular expectations to ensure the accuracy and authenticity of the data measurement. The mean ADC and FA value were calculated for statistical analysis. Images with obvious artifacts will be excluded.In addition, the rats with positive urine albumin should also be eliminated.

### Histopathology

After MR scanning, all animals were sacrificed by intraperitoneal injection of 100mg/kg pentobarbital (Dalian Meilun Biotechnology Co., LTD, China). The bilateral kidneys were resected, and tissue samples were sliced using histological microtome (Vicker Science education instrument Co., LTD, China), fixed with 4% formalin (Beijing Lanjieke Technology Co., LTD, China), and embedded in paraffin for hematoxylin and eosin (HE) and periodic acid Schiff (PAS) staining. The sections were examined with light microscopy (NIKON Eclipse ci, Japan) and the images of histopathology were obtained with magnification of 400 ×.

### Statistical Analysis

Statistical analysis was performed by using SPSS17.0 statistical software (SPSS, IBM Corp., Armonk, NY). *P* values < 0.05 were considered to be indicative of significant differences. All quantitative parameters were tested by normal distribution and homogeneity tests of variance. The mean values of laboratory parameters, ADC, and FA of the two groups were calculated and analyzed by the independent two-sample t-test.

## Results

### Summary of The General Condition and Biochemistry

According to the classification criteria of Mogensen (14), fourteen successful and surviving DN rats met the diagnosis of preclinical diabetic nephropathy.28 kidneys from 14 rats in the DN group and 20 kidneys from 10 rats in the NC group were resected. Streptozotocin-induced diabetes resulted in decreased weight and elevated serum glucose in the DN rats relative to those in the controls (*P* = 0.002 and *P* < 0.001, respectively). The urinary output was more than fourfold higher in the DN rats than in the controls (*P* < 0.001). There was no significant difference in serum creatinine between the two groups (*P* > 0.05). In the DN group, the urinary albumin levels at 2, 4, and 8 weeks were higher than those of the animals in the NC group, but all had values less than 20 ug/min, and there was a statistical difference between the two groups (*P ≤* 0.001; **Table 1**). According to the classification criteria of Mogensen, 14 rats (28 kidneys) met the stage 2 diabetic nephropathy.

**Table 1:** General condition and biochemistry (x̄ ± s).

	NC group	DN group	t/p
**weight(g)**	528.8 ± 20.5	373.7 ± 45.5	3.923/0.002
**serum glucose(mmol/l)**	5.98 ± 1.37	27.46 ± 3.14	-14.283/0.000
**serum creatinine(umol/l)**	72.92 ± 15.47	85.41 ± 24.91	-0.489/0.634
**24h urine volume(mL)**	11 ± 1.58	43.25 ± 6.78	-12.909/0.000
**urinary albumin at 2 week (ug/min)**	4.89 ± 0.84	14.43 ± 3.91	-5.293/0.000
**urinary albumin at 4 week (ug/min)**	6.11 ± 1.63	16.8 ± 5.27	-4.343/0.001
**urinary albumin at 8 week (ug/min)**	8.2 ± 1.03	15.50 ± 2.85	-6.593/0.000

### Pathology

**Figure 1:**
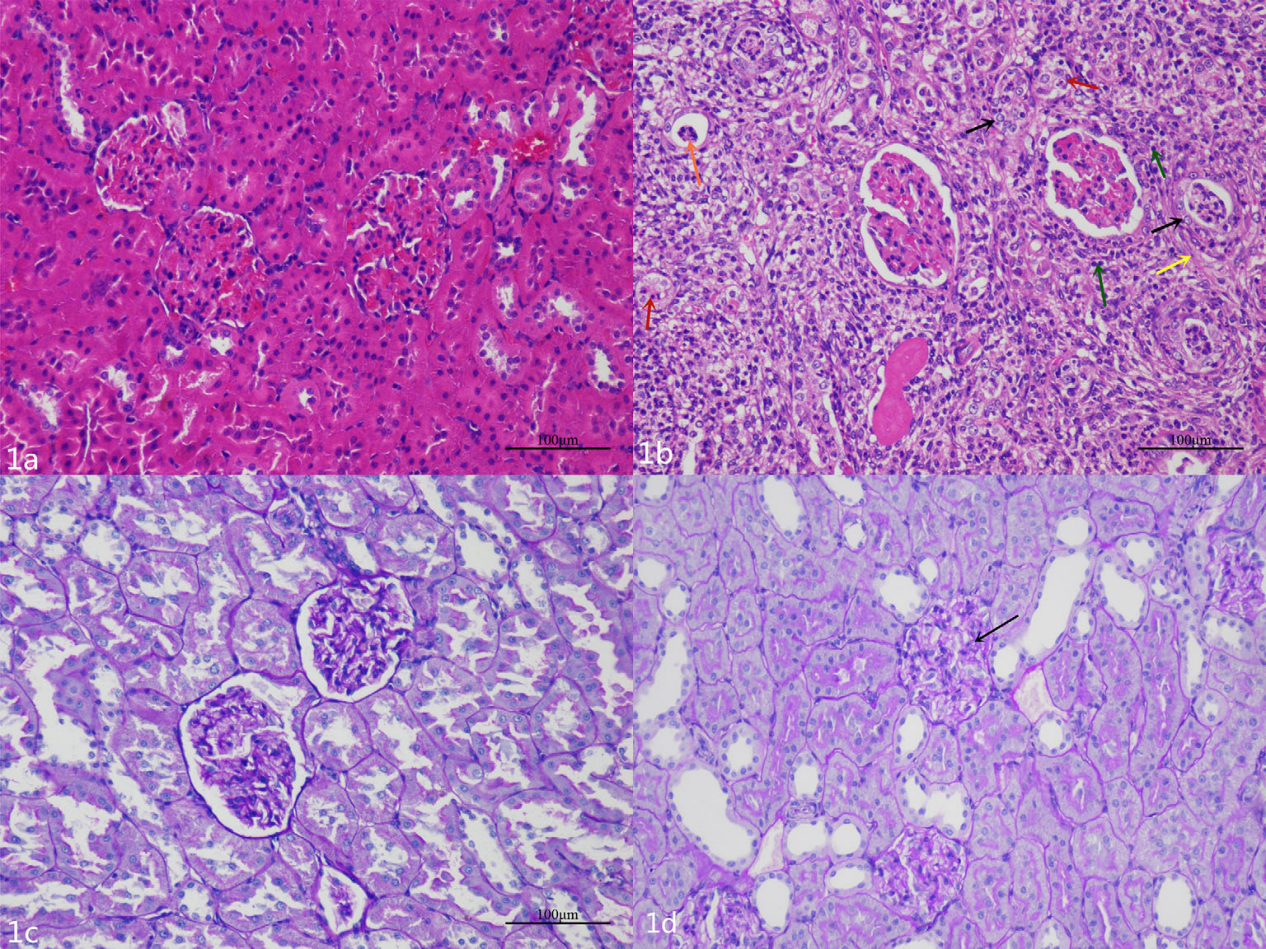
**(A)** The glomeruli and tubules of the NC group are normal. **(B)** Hematoxylin and eosin staining shows that there are different degrees of renal pathological injury in the DN rats. The structure of the renal tissue is disordered, the renal tubular structure was not clear, a large number of renal tubular epithelial cells are swollen, and the cytoplasm is loose (black arrow); a small number of renal tubular epithelial cells are necrotic, and the nucleus is deeply stained or fragmented (red arrows).The renal tubular interstitial connective tissue is hyperplasic (yellow arrow) and accompanied by a small amount of inflammatory cell infiltration (green arrows); necrotic cell fragments can be seen in some renal tubules (orange arrow). **(C)** The renal basement membrane in the NC group is normal. **(D)** The basement membrane is slightly thickened and wrinkled (black arrow), and the mesangial cell is mildly hyperplasic in the DN group.

HE and PAS staining (**Figures 1A, C**, respectively) showed that there were no significant abnormal changes in renal glomeruli and tubules in the NC group. In the DN group, HE staining (**Figure 1B**) showed no obvious abnormality in the structure and morphology of the glomeruli. The structure of renal tubules was unclear or disappeared. The renal tubular epithelial cells were swollen, and the cytoplasm was loose. Some of the tubular epithelial cells were necrotic, and the nucleus showed pyknosis and deep staining or fragmentation. Partial tubular interstitial connective tissue hyperplasia with inflammatory cell infiltration was observed, and a portion of the renal tubular lumen showed necrotic cell fragments. PAS staining (**Figure 1D**) showed that the glomerular basement membrane of the DN rats was slightly thickened, and the PAS-positive area was increased.

### MR Imaging

**Figure 2:**
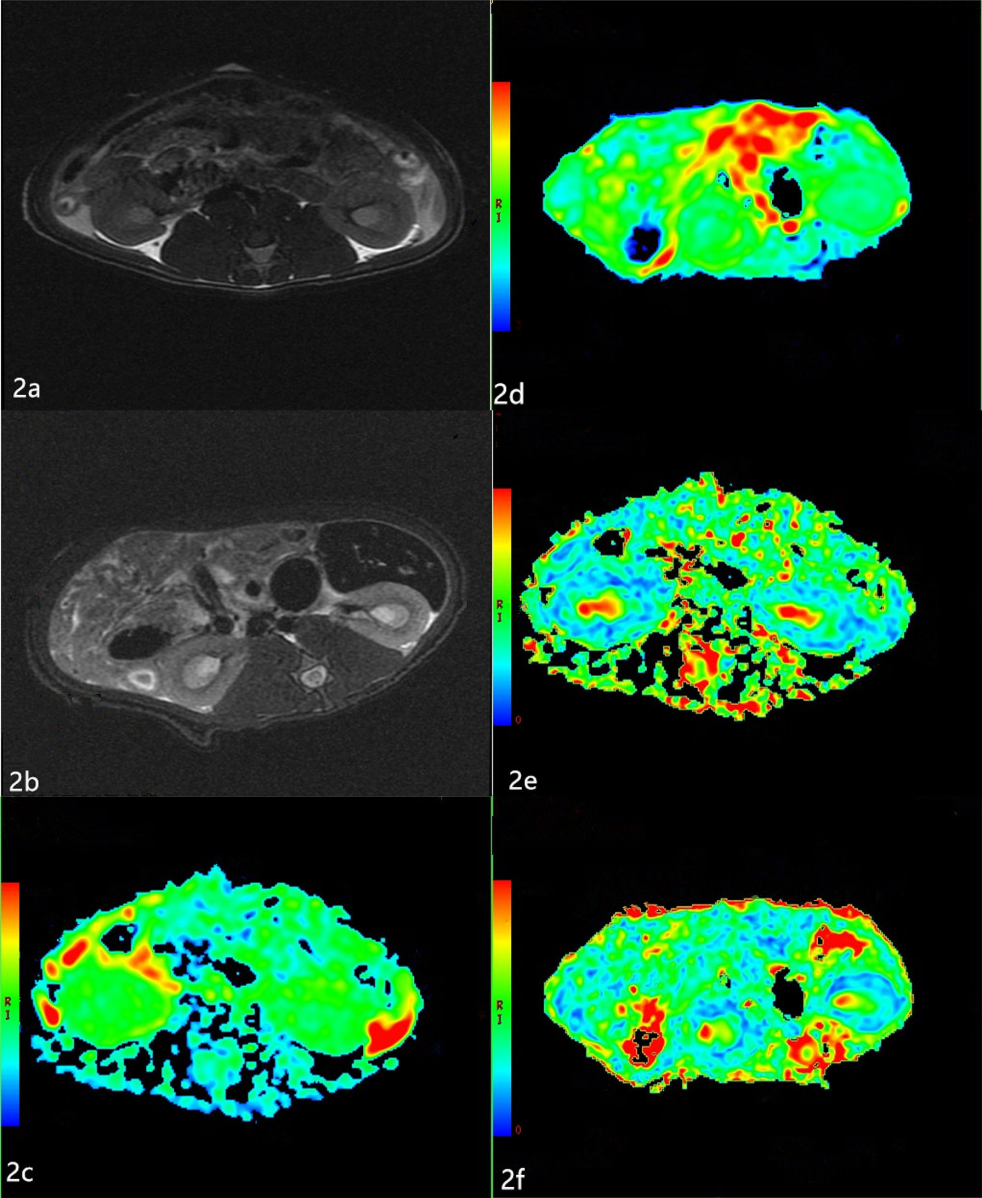
Apparent diffusion coefficients (ADC) and Fractional anisotropy (FA) maps of the two groups **(A, B)** The T2-weighted images of the two groups. T2-weighted images show no obvious changes in the morphology and structure of the kidneys between the two groups, and there is clear differentiation between the renal cortex and medulla. **(C, D)** The ADC maps of the NC group and DN group. The boundary of the cortex and medulla are clear in the NC group. The resolution of the cortex and medulla of the kidneys in the DN group is indistinct. **(E, F)** The FA maps of the NC group and DN group. The boundary of the cortex and medulla are clear, but the color resolution of the renal cortex and medulla is lower in the DN group than in the NC group.

T2-weighted images showed no obvious differences in the morphology and structure of the kidneys between the two groups, and there was clear differentiation between the renal cortex and medulla. Examples of ADC and FA maps in the NC group and DN group are shown in **Figure 2**.

The ADC values of the renal cortex and medulla were significantly higher in the DN rats than in the NC animals (*P* < 0.05; **Table 2**). The FA values of the renal cortex and medulla were significantly lower in the DN group than in the NC group (*P* < 0.05; **Table 3**).

**Table 2:** The ADC value of renal cortex and medulla (×10 ^-3^ mm^2^/s).

Group	Number of kidneys	Cortex	Medulla
**NC**	20	1.52 ± 0.28 (95%CI 1.37˜1.67)	1.35 ± 0.13 (95%CI 1.28˜1.42)
**DN**	28	1.75 ± 0.35 (95%CI 1.62˜1.89)	1.53 ± 0.3 (95%CI 1.41˜1.64)
***t/p***		-2.227/0.028	-2.223/0.032

**Table 3:** The FA value of renal cortex and medulla.

Group	Number of kidneys	Cortex	Medulla
**NC**	20	0.26 ± 0.06 (95%CI 0.22˜0.29)	0.30 ± 0.04 (95%CI 0.28˜0.32)
**DN**	28	0.21 ± 0.05 (95%CI 0.19˜0.23)	0.25 ± 0.06 (95%CI 0.23˜0.27)
***t/p***		2.345/0.024	3.129/0.003

## Discussion

Generally, DN is categorized into five stages on the basis of the Mogensen (14) criteria: hyperfiltration stage (stage 1), normal albuminuria stage (stage 2), microalbuminuria stage (stage 3), clinical DN stage (stage 4), and end-stage renal failure (stage 5). Among them, stages 1 and 2 comprise the preclinical stage. Three days after modeling, all animals in the DN group developed hyperglycemia, polyuria, and emaciation. The renal pathological damage in the DN rats, such as tubule disappearance, tubule epithelial cell swelling, and inflammatory cell infiltration, were observed, which were in accordance with the preclinical DN stage.

DTI is a promising technique to non-invasively evaluate water molecule diffusion features in the renal parenchyma. Compared with diffusion-weighted imaging, DTI can provide more functional parameters, such as FA, other than ADC. FA is able to provide information about the diffusion direction and its degree at the same time. It has been shown that the FA values of the renal cortex and medulla are reduced in DN patients with or without microalbuminuria relative to those in normal healthy volunteers (6, 15). Yan (5) found that the cortical FA value was significantly lower in early DN rats than in the NC group. The present study showed that the FA values of the renal cortex and medulla were lower in the DN group than in the NC group. These results indicated that the directed diffusion of water molecules in early DN was restricted. The pathological mechanism of FA reduction is not clear. We found that even in the early stage of DN, the kidney showed pathological changes, such as swelling or necrosis of tubular epithelial cells, proliferation of interstitial connective tissue with varying degrees of inflammatory cell infiltration, and filling of cell fragments in the renal tubule lumen, which are in good agreement with those of previous studies (16, 17). Hueper et al. (18) found that reduction of renal FA was significantly and negatively correlated with the extent of renal pathologies, such as glomerulosclerosis, interstitial fibrosis, and tubular damage. Cheung et al. (19) believed that tubular dilation removes part of the directionality of diffusivity along the tubules and therefore explains FA reduction. In addition, at the early stage of DN, the cellular debris congests the tubules, which also weakens the directional diffusion of water molecules (18, 20). We also found that the FA value was lower in the renal cortex than in the medulla in both groups, which was consistent with other study findings (15, 21, 22). This finding may be related to the anatomical characteristics of the kidney. The renal medulla is composed of collecting ducts and some microvascular structures, which arrange radially in the direction of the pelvic cavity. Because of the anatomical characteristics of the renal medulla, the movement of water molecules is more complex in the medulla than in the cortex. But unlike most other studies,our study focuses on preclinical DN, although the urine microalbumin is within the normal range, the renal function has changed at this time. It is possible to provide valuable imaging information for clinical treatment of DN as early as possible.

The diffusion of water molecules is influenced by the water content of the kidney, random motion of water molecules, microcirculation blood flow perfusion, glomerular filtration, reabsorption and secretion in renal tubules, and cell structure (23). In this study, we found that the ADC values of renal cortex and medulla significantly increased in the DN group relative to those in the NC group. These changes may be related to the physiological function of the kidney, which maintains the balance of acid–base and water–salt metabolism mainly through the filtration of glomeruli and reabsorption and secretion in renal tubules. In the preclinical stage of DN, with the increase of renal blood perfusion and the glomerular filtration rate, the amount of water molecules is larger than that in healthy kidneys. However, in preclinical DN, the degree of renal pathological damage is slight, which will not affect the formation and resorption of urine. The diffusion limitation of water molecules caused by renal pathological damage is not obvious at this stage. Therefore, the effect of renal hyperperfusion on the ADC value is greater than that of renal pathological damage, which has been reported by other researchers (5, 24). Cakmak et al. (25) found that the ADC values in patients with stage 3 DN were significantly lower than that in healthy people and they were more obvious in patients with stage 4 and 5 DN. A reduction in the glomerular filtration rate reflects reduction of hyperfiltration. In this way, a lower rate of water transfer across the interstitial space leads to reduced diffusion. When overt proteinuria occurs, histopathological damage is often far advanced. Progressive glomerulosclerosis and tubulointerstitial fibrosis may also restrict water diffusion (26). These effects may oppose the effect of hyperfiltration.

Our study had some limitations. First, only preclinical DN models were included, so we could not compare results with those of stage 3, 4, and 5 DN. Second, this study did not analyze the correlation between DTI parameters and pathological damage. Thirdly, there may be some measurement errors because ROIs were drawn manually, and STZ may has certain nephrotoxicity, which may cause acute kidney injury in rats (27), and interfered with renal changes.

## Conclusion

In the preclinical DN model, renal cortical and medullary ADC values were significantly increased and FA values were significantly reduced relative to those in healthy animals. DTI might serve a potential role in early, non-invasive, and quantitative diagnosis, and therapy evaluation of DN.

## Data Availability Statement

The raw data supporting the conclusions of this article will be made available by the authors, without undue reservation.

## Ethics Statement

The animal study was reviewed and approved by the ethics committee of Chengdu First People’s Hospital.

## Author Contributions

All authors contributed to the study conception and design. Data curation, XH and YW. Formal analysis, YW. Investigation, BP, MK, YY and WL. Methodology, XH, BP, MK, YY and WBL. Project administration, WBL and YW. Writing – original draft, XH. All authors contributed to the article and approved the submitted version.

## Conflict of Interest

The authors declare that the research was conducted in the absence of any commercial or financial relationships that could be construed as a potential conflict of interest.

## Publisher’s Note

All claims expressed in this article are solely those of the authors and do not necessarily represent those of their affiliated organizations, or those of the publisher, the editors and the reviewers. Any product that may be evaluated in this article, or claim that may be made by its manufacturer, is not guaranteed or endorsed by the publisher.
